# 16S rRNA Amplicon Sequencing Demonstrates that Indoor-Reared Bumblebees (*Bombus terrestris*) Harbor a Core Subset of Bacteria Normally Associated with the Wild Host

**DOI:** 10.1371/journal.pone.0125152

**Published:** 2015-04-29

**Authors:** Ivan Meeus, Laurian Parmentier, Annelies Billiet, Kevin Maebe, Filip Van Nieuwerburgh, Dieter Deforce, Felix Wäckers, Peter Vandamme, Guy Smagghe

**Affiliations:** 1 Laboratory of Agrozoology, Department of Crop Protection, Faculty of Bioscience Engineering, Ghent University, Coupure Links 653, B-9000, Ghent, Belgium; 2 Laboratory of Pharmaceutical Biotechnology, Faculty of Pharmaceutical Sciences, Ghent University, Ottergemsesteenweg 460, B-9000, Ghent, Belgium; 3 Biobest NV, Ilse Velden 18, B-2260, Westerlo, Belgium; 4 Laboratory of Microbiology, Department of Biochemistry and Microbiology, Faculty of Sciences, Ghent University, K. L. Ledeganckstraat 3, B-9000, Ghent, Belgium; University of Camerino, ITALY

## Abstract

A MiSeq multiplexed 16S rRNA amplicon sequencing of the gut microbiota of wild and indoor-reared *Bombus terrestris* (bumblebees) confirmed the presence of a core set of bacteria, which consisted of *Neisseriaceae* (*Snodgrassella*), *Orbaceae* (*Gilliamella*), *Lactobacillaceae* (*Lactobacillus*), and *Bifidobacteriaceae* (*Bifidobacterium*). In wild *B*. *terrestris* we detected several non-core bacteria having a more variable prevalence. Although *Enterobacteriaceae* are unreported by non next-generation sequencing studies, it can become a dominant gut resident. Furthermore the presence of some non-core lactobacilli were associated with the relative abundance of bifidobacteria. This association was not observed in indoor-reared bumblebees lacking the non-core bacteria, but having a more standardized microbiota compared to their wild counterparts. The impact of the bottleneck microbiota of indoor-reared bumblebees when they are used in the field for pollination purpose is discussed.

## Introduction

Bumblebees play an important role in pollination networks, providing an essential ecological service to maintain plant diversity [1, 2] and a commercial service for many agricultural crops [3]. To date there are major concerns towards a global decline in the natural pollinator diversity [4]. There is growing evidence of dramatic declines of bumblebees in Europe and other parts of the world [2, 5, 6]. Multiple causes have been recognized including forage and habitat loss [7], pesticide use [8], competition with non-native bees, pathogen emergence [9] and others [10]. The trend of decline can be seen worldwide, but the impact of different drivers is diverse across geographic locations [11]. Some of these drivers of bumblebee decline could also influence the host microbial community (microbiota). A normal gut microbiota is essential for health and a disrupted gut microbiota (dysbiosis) can invoke a range of diseases [12]. In bumblebees too, intestinal dysbiosis or increased richness of non-core bacteria was associated with higher incidence of infection with the gut parasite *Crithidia bombi*[13, 14]. Indeed a particular set, called the core gut microbiota [15, 16], is specifically associated with bumblebees and honeybees, while absent in solitary bee species [16]. Koch et al. [17] performed a cospeciation study sequencing a 16S rRNA library of two core bacteria, *Snodgrassella alvi* and *Gilliamella apicola*. They showed that the resulting bacterial strains per species are rather structured over bumblebee hosts than over geographic locations, thereby underlining an association between the host and their bacteria, predisposing them to possibly evolve a functional dependence.

The microbiota of insects is not only linked with protection against parasites; a vast variety of host-beneficial functions has been reported, including food digestion and detoxification [17]. Therefore stressors acting upon the gut microbiota could substantially weaken the bumblebee colony, even further deteriorating their current threatened status.

In this study, we focus on one host species, the buff-tailed bumblebee *Bombus terrestris*, and perform a MiSeq deep sequencing with Illumina and MID technology (multiplex identifier). We compared the gut microbiota of 24 wild buff-tailed bumblebee workers originating from three different environments in Belgium, with *B*. *terrestris* workers from an enclosed mass rearing system for multiple generations (Biobest). This comparison will allow us to describe how natural the microbiota of these intensively indoor-reared bumblebees really is. Furthermore the identification of bacteria in indoor-reared bumblebees provides us insights in their host association, because their hosts have been separated from the typical bee-environment and thus excluded of potential horizontal transmission of bee environment-associated bacteria.

## Materials and Methods

### Ethics Statement

No national permissions were required to collect samples from the public lands in the locations of Flanders (Belgium). We did not sample any endangered or protected species.

### Specimens

24 wild *B*. *terrestris* workers were collected in three different environments, as described in details in Parmentier et al. [18]. Location W1 is an urban area with patchy green areas, location W2 is a rural area, while location W3 is an urban area with low abundance of green area. Sampling was performed within the same week in June 2012.

Indoor-reared bumblebees were obtained from the bumblebee mass-breeding company Biobest (Westerlo, Belgium). We used 14 workers and each worker was collected from a different colony, each containing approximately 25 workers and one queen.

### Illumina sequencing and taxonomic identification

The whole gut, including the crop, was dissected and stored at -20°C. The gut was crushed in a 170 μL lysozyme solution (100 mg/ml) and DNA was extracted according to Meeus et al. [19]. The V4 region of the rRNA was amplified in triplicate, using the composite 515F and 806R primers designed by Caporaso et al. [21]. The composite primers contain the 16S primer sites, a different nucleotide barcode (on the 806R primer) for each sample and the Illumina adapters sequences that are necessary for the bridge amplification on the Illumina MiSeq flow cell [20]. Amplicons were normalized after quantification of the amount of double stranded DNA with the Quant-iT PicoGreen dsDNA reagent (Life Science technologies) on an Infinite M200 microplate reader (Tecan). Samples were mixed at equimolar concentrations and purified using the E.Z.N.A. Cycle Pure Kit and further concentrated with Amicon Ultra-0.5 Centrifugal Filter Device. The equimolar pool was denatured and diluted following Illumina protocols to produce a 8 pM sequencing library. Twenty percent denatured Illumina PhiX Control V3 library was admixed to increase sequence diversity of the final library. Cluster generation and 2x150 paired-end sequencing was performed in one Illumina Miseq flowcell using an Illumina MiSeq Reagent Kit v2. Custom sequencing primers were added to the primers in the kit at a final concentration of 0.5 μM because the adapters on the composite primers do not contain the standard sequencing primer sites. Basecalling and primary quality assessments and de-multiplexing were performed using Illumina’s Basespace genomics cloud computing environment.

The complete dataset contained 3,428,218 demultiplexed paired-end reads flagged as ‘pass filter’ by the Basespace analysis. Sequences were analyzed with the mothur software v.1.31.1 [21], mainly following the standard operating procedure available on http://www.mothur.org/wiki/MiSeq_SOP date 4 March 2014 [22]. Before clustering sequences into OTUs (operational taxonomic units), the complexity was reduced by retaining unique sequences shorter than 275 base pairs and without any ambiguous base pairs. This resulted in 1,747,090 total reads of which 271,532 were unique sequences. Denoising was performed by preclustering all sequences with 1 mutation on 100 base pairs. The UCHIME algorithm [23], with the abundant sequences as reference, identified approximately 9% of the unique sequences as possible chimeras. Furthermore, a large fraction of unique sequences (121,474) only occurred once; these were removed to reduce file complexity, resulting in 2,128 unique sequences of which 4 were excluded because they did not belong to the bacteria domain. Although the amount of unique sequences dropped from 271,532 to 2,218, the dataset still contained the majority of the reads (i.e.1,520,753).

Calculating the distance matrix and clustering with a 0.03 cutoff level resulted in 111 OTUs. A two fold strategy was performed to exclude sequencing errors: first, only OTUs having more than 0.5% of the sequence reads in any sample were kept, and in addition, all sequences not yet reported in bumblebees or honeybees were confirmed by conventional PCR with sequence-specific primers. This procedure resulted in 22 OTUs representing 99.7% of the total reads. A list of all bacterial taxa and those excluded from our analysis is provided in a S1 Dataset.

The taxonomic identity of each OTU was revealed by alignment of each sequence with the Bacterial SILVA SEED database. This database (training set) was supplemented with host specific sequences (i.e. host *Apis* or *Bombus*) to improve classification [24]. In order to reduce the size of the training set we only included the 805 sequences which were the representative sequence of 99% identity clusters. The identity of each OTU was confirmed by BLASTn of the representative sequences. The get.oturep command in mothur retrieves the representative sequences based on the distance matrix of sequences within one OTU. All representative sequences are provided in a S2 Dataset. All sequences confirmed with OTU specific primers were aligned with their representative sequences and identified with the BLASTn algorithm against the non-redundant nucleotide collection and deposited at GenBank (KM030545 until KM030553). Raw Illumina data reads are submitted to the SRA database of Genbank under accession ID SRP050540.

### Verification of new OTUs

To confirm the representative OTU sequence obtained after Illumina sequencing a semi-nested PCR with a universal Eub8F or 984yR primer combined with a OTU specific primer was performed (see Table 1). This proved that their presence is not an artifact of random sequencing errors or chimera formation. All OTU specific primers were designed with primer3. A sequence was assigned to a specific OTU if there was a 100% sequence identity with the overlapping sequence of the representative OTU. The external PCR had 25 cycles with an annealing temperature of 53°C. The 50 times diluted PCR product underwent an internal touch-down PCR, with 5 cycles starting from 52°C towards 50°C, followed by an extra 20 cycli at 50°C.

**Table 1:** OTU specific primers combined with a universal 16S rDNA primer (Eub8F or 984yR).

Target	Forward primer	Reverse primer
Gamma-E1	TGTCAAGTCGGATGTGAAAT	984yR GTAAGGTTCYTCGCGT
Gamma-E1	Eub8F AGAGTTTGATCMTGGCTCAG	TCACATCCGACTTGACAGAC
Gamma-E2	ACTGCATTTGAAACTGGTCA	984yR
Gamma-E2	Eub8F	ATGCAGTTCCCAAGTTAAGC
Lacto5	Eub8F	CTGTCCTCTTCTGCACTCAA
Firm-S	Eub8F	TCCTGCACTCAAGTCTACCA
Firm-E	Eub8F	GTCTCCCAGTTTCCAATGAC
Lacto3	Eub8F	AGTTTCCACTGCACTTCCTC
Firm-B	Eub8F	GTCTCCCAGTTTCCAATGAC
Gamma_P	Eub8F	CTAGCTTGCCAGTTTTGGAT
Burk	Eub8F	CACTCCAGCTATGCAGTCAC
*Actinomycetales*	Eub8F	GCTGTGAGTTTTCACAAACG

### Identification of sisters

Sampling of multiple bumblebees from a certain location can contain several sisters. The presence of sisters within a selected locality can influence the comparison of the microbiota among locations. To examine the family relationships, we genotyped the 24 wild bumblebees with 10 microsatellite loci as described in [25]. Bumblebee DNA extraction, PCR amplification, capillary electrophoreses and allele scoring were made following the protocol as described in [26]. For identification of the possible sisters, we used the program Colony 2.0 [27] employing corrections for genotyping errors (5% per locus).

### Characterization of gut microbiota

Samples were normalized to the smallest number of reads for a given sample (n = 16,426). The normalized shared files, generated in the standard operating procedure of the mothur software, were used to generate the diversity calculators and associations, i.e. rarefaction.shared, summary.shared and otu.association. Bacterial evenness (e) was calculated as e = H/ lnS, where H is the Shannon index and S is the number of OTUs [28, 29]. The normalized bacterial abundance is the total number of bacterial reads after normalization. This value cannot be regarded as an absolute quantity, since the total amount of bacteria can differ in different bumblebee guts [13] and will later on be referred to as relative abundance. Differences of the diversity calculators were determined by the non-parametric Kruskal-Wallis test in SPSS comparing the specimens from the 3 wild locations and the specimens from a commercial breeding facility.

A multivariate approach with generalized linear models (GLM) in R was followed to compare the relative abundance of different OTUs between the microbiota of indoor-reared and wild bumblebees. Again location was chosen as the dependent variable. For multivariate data GLM outperforms distance-based methods in terms of power, not missing low abundant species effects [30]. Count data with high abundance in combination with zero values often have a negative binomial distribution, with a mean variance plot tending to be quadratic [31]. The S1 Fig shows the mean-variance plot and the residual vs fits plot showing least pattern for a negative binomial distribution, therefore we ran the manyglm command with family = neg.binom in the mvabund package [32]. For post-hoc testing again Kruskal-Wallis test were performed in SPSS. In order to improve visualization of the abundance data was transformed log(y/a + 1) with a the minimum possible non-zero abundance, this reduces the dominance of few values with high abundance [33].

Nonmetric multidimensional scaling (NMDS) was used to visualize differences in the bacterial community based on a Bray-Curtis similarity matrix of the square root transformed relative abundance of the different OTUs per sample. Clusters of similarity was based on the Bray-Curtis similarity matrix (Primer6 version 6.1.10). Differences in similarity between sisters and non-sister bumblebees were calculated by analysis of similarities (ANOSIM). It calculates a global R statistic which lies between −1 and +1, with high absolute values indicating a large degree of discrimination among groups.

## Results

### The characteristic phylotypes of wild Bombus terrestris

The gut microbiota of 24 wild bumblebee workers foraging in three different locations (W1–W3) was analyzed. In total we identified 23 different OTUs, after OTU picking with 97% similarity. Table 2 gives an overview of all the OTUs identified. Their nomenclature is based on the bacterial family to which they belong supplemented with previous nomenclature to show similarity with other studies. The new OTUs were confirmed by PCR with OTU-specific primers (see Table 1). For the two closely related OTUs representing *Burkholderiales* and the three closely related OTUs representing *Actinomycetales*, we only identified one bacterial sequence. Also the BifidoX OTU could not be confirmed with PCR because of primer cross reactivity with Bifido1, 2 or 3.

**Table 2:** Taxonomic identification of OTUs and their closest match in GenBank.

*Phylum* *Order or Family*	Name used here (other names)	Matching basepairs/total basepair first match previously identified in corbiculate bees (non-deep sequencing data)	Association	First match not found in corbiculate bees (non-deep sequencing data)	References
*Alphaproteobacteria*	Alpha1	253/253 JQ673261	Gut *Apis*		[34, 46]
*Betaproteobacteria*					
*Neisseriaceae*	*Snodgrassella* (beta)	253/253 *Snodgrassella alvi*	Gut *Apis* and *Bombus*		[16, 34, 47]
*Burkholderiales*	Burk1, 2^$^			524/532 JQ658329^$^ 492/533 HM111030* 470/535 HM108635**	
*Gammaproteobacteria*					
*Orbaceae*	*Gilliamella* (Gamma-1)	253/253 *Gilliamella apicola*	Gut *Apis* and *Bombus*		[16, 34, 47]
*Enterobacteriaceae*	Gamma-E1			870/881 CP003938 793/843 AJ971871**	
*Enterobacteriaceae*	Gamma-E2			794/794 JX860524 726/785 AJ971871**	
	Gamma2	253/253 HM215025	Gut *Bombus*	245/253 NR118490***	[16, 48]
*Pseudomonadaceae*	Gamma-P			526/527 KC502873	
*Bacteroidetes*	*Bacteroidetes*	253/253 JQ388908	Gut *Bombus* and *Apis*		[15]
*Firmicutes*					
*Lactobacillaceae*	Lacto1-Firm5 (Firm-5. *Lactobacillus* (VI))	253/253 HM215048	Gut *Bombus*		[15, 16, 34, 47]
*Lactobacillaceae*	Lacto2-Firm4 (Firm-4)	253/253 KJ078645	Gut *Bombus* queen		[15, 16, 34, 47]
*Lactobacillaceae*	Lacto3	555/573 HM534759	Crop *Apis*		
*Lactobacillaceae*	Lacto4 (*Firmicutes* (V))	253/253 JQ388900			[15]
*Lactobacillaceae*	Lacto5	581/581 EU753703	Crop *Bombus*		[49]
*Streptococcaceae*	Firm-S			564/564 KJ186939	
*Enterococcaceae*	Firm-E			402/402 KJ156978 379/402 AJ971886**	
*Bacillaceae*	Firm-B	547/550 AJ971921	Gut *Bombus*		[49]
*Actinobacteria*					
*Bifidobacteriaceae*	Bifido1 (Killer group 1. *Bifidobacterium actinocoloniiforme*)	253/253 FJ858735	Gut *Bombus*		[50, 51]
*Bifidobacteriaceae*	Bifido2 (Killer group 2)	253/253 FJ858732	Gut *Bombus*		[50, 51]
*Bifidobacteriaceae*	Bifido3 (Killer group 3)	253/253 FJ858733	Gut *Bombus*		[50, 51]
*Bifidobacteriaceae*	BifidoX			247/253 JQ354974	
*Actinomycetales*	Myc1, 2, 3^$^			491/491 KC128891^$^ 474/488 AJ971863**	

All OTUs previously identified in corbiculate bees by non-deep sequencing techniques have their first blast hit in the third column, others in the fifth column.

*abomen of wild bee Halictus patellatus;

** Bombus sp.;

*** Frischella perrara from Apis mellifera

^$^ only one of the OTUs was confirmed with OTU specific primers

### Related foraging bumblebee workers have a more similar microbiota

**Fig 1:**
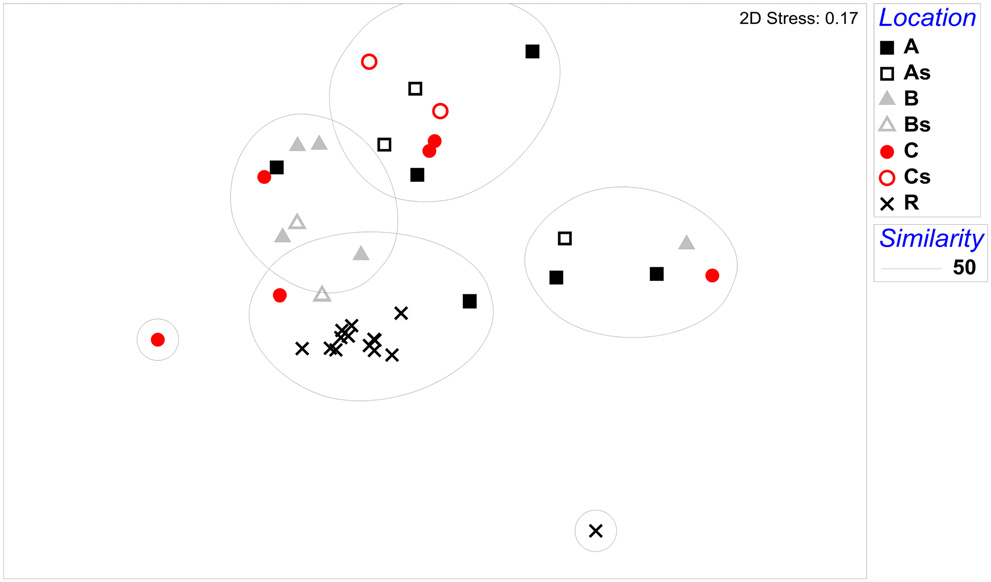
NMDS non-metric multidimensional scaling of the bumblebee microbiota of wild (location W1, W2 and W3) and indoor-reared (R) *Bombus terrestris*. The open symbols are sister specimens from each location, also annotated by the extra letter s after the location indicator. The circles group samples with a higher similarity than 50% based on the Bray-Curtis similarity matrix.

We sampled in three locations (W1–W3). Rarefaction curves reaching a plateau (S2 Fig) illustrate that 16,000 sequence reads per sample and 7 specimens per location had sufficient depth. Microsatellite analysis revealed 3 possible sisters at location W1, 2 at location W2, and again 3 at location W3. Although sisters can have large variation in their microbiota and fall within different regions of the non-metric multidimensional scaling plot (Fig 1; open symbols represent sisters), the similarity among sisters is higher (ANOSIM, R = 0.55, *P* = 0.01). Therefore we did not automatically exclude sisters for further data analysis, but we only excluded a sister if her microbiota showed more than 70% similarity with an earlier sampled sister.

### The microbiota of intensively indoor-reared B. terrestris is a subset of the wild microbiota

The microbiota of 14 indoor-reared bumblebee workers revealed 9 OTUs. Indoor-reared bumblebees contained 2 OTUs which we did not retrieve in the wild bumblebees, however these two OTUs (i.e. Gamma-2 and Firm-B) only represented 0.8% of the bacterial sequence reads in reared bumblebees. The lower number of OTUs is not a consequence of a lower number in specimens, as each sampling location of wild bumblebees had more OTUs (S2 Fig).

**Fig 2:**
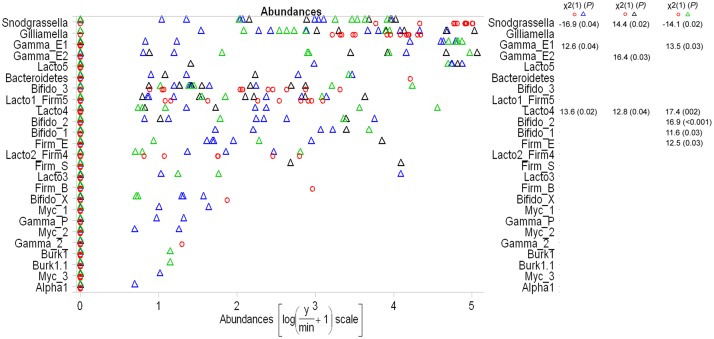
The normalized and transformed abundance of the different OTUs of wild and indoor-reared *Bombus terrestris*. The wild location A, B, C are represented by a blue, black and green triangle, respectively. The indoor-reared bumblebees are represented a line lower by a red circle. On the right side the pairwise Kruskal-Wallis post-hoc test are given for each OTU, but only the significant ones (*P* adjusted < 0.05). No significant values were found between wild locations.

All other bacterial OTUs (representing 99.2% of the bacterial sequence reads) of indoor-reared bumblebees were also retrieved in wild species, although in wild bumblebees these OTUs only represent 40.2% of the total bacterial reads. Fig 2 gives an overview of the relative abundance of all OTUs in the three sampling locations (W1, W2 and W3), compared to the relative abundance in indoor-reared bumblebees. GLM support a significant difference within these locations (Dev = 173.6, *P* = 0.001), the univariate test are given in the S1 Table. Significant pairwise post-hoc tests (Kruskal-Wallis) of all locations per OTU are shown in Fig 2. We only found differences between the indoor-reared bumblebees and specimens collected in the wild. All core bacteria, as defined by Cariveau et al. [13], were present in the indoor-reared bumblebees, except the *Alphaproteobacteria*. Of the latter, only Alpha1 was present in one single wild specimen of our dataset with a low relative abundance. The relative mean abundance of each OTU and the prevalence in wild and indoor-reared bumblebees is given in Table 3.

**Table 3:** Normalized mean abundance of each OTU and its prevalence in its host *Bombus terrestris*.

*Phylum* *Order or Family*	Name used here bold = appear in reared specimens	Wild specimens		Reared specimens			
		Normalized mean abundance (%)	Present in host (%)	Normalized mean abundance (%)	Present in host (%)	Core in honeybees	Core in *Bombus terrestris*
*Alphaproteobacteria*	Alpha1	0.02	5	0.00	0	Alpha1, Alpha2.1 and Alpha2.2	
*Betaproteobacteria*							
*Neisseriaceae*	***Snodgrassella***	10.66	82	57.22	93	*Snodgrassella*	*Snodgrassella*
*Burkholderiales*	Burk1	0.04	5	0.00	0		
*Burkholderiales*	Burk2	0.06	5	0.00	0		
*Gammaproteobacteria*							
*Orbaceae*	***Gilliamella***	19.19	91	23.63	93	*Gilliamella*	*Gilliamella*
*Enterobacteriaceae*	Gamma-E1	16.14	36	0.00	0		*Enterobacteriaceae*
*Enterobacteriaceae*	Gamma-E2	14.43	50	0.00	0		*Enterobacteriaceae*
	**Gamma2**	0.00	0	0.10	7	*Frischella*	
*Pseudomonadaceae*	Gamma-P	0.05	5	0.00	0		
*Bacteroidetes*	**Bacteroidetes**	1.78	27	9.04	14		
*Firmicutes*							
*Lactobacillaceae*	**Lacto1-Firm5**	3.24	68	3.72	64	Lacto1-Firm5	Lacto1-Firm5
*Lactobacillaceae*	**Lacto2-Firm4**	0.85	23	1.42	43	Lacto2-Firm4	*Lactobacillaceae*
*Lactobacillaceae*	Lacto3	1.60	18	0.00	0	*Lactobacillaceae*	*Lactobacillaceae*
*Lactobacillaceae*	Lacto4	3.68	41	0.00	0	*Lactobacillaceae*	*Lactobacillaceae*
*Lactobacillaceae*	Lacto5	14.06	32	0.00	0	*Lactobacillaceae*	*Lactobacillaceae*
*Streptococcaceae*	Firm-S	1.67	9	0.00	0		
*Enterococcaceae*	Firm-E	2.06	32	0.00	0		
*Bacillaceae*	**Firm-B**	0.00	0	0.69	7		
*Actinobacteria*							
*Bifidobacteriaceae*	Bifido1	2.90	27	0.00	0		*Bifidobacteriaceae*
*Bifidobacteriaceae*	Bifido2	3.22	45	0.00	0		*Bifidobacteriaceae*
*Bifidobacteriaceae*	**Bifido3**	3.92	77	3.91	79		Bifido3
*Bifidobacteriaceae*	**BifidoX**	0.29	23	0.22	7		*Bifidobacteriaceae*
*Actinomycetales*	Myc1	0.07	5	0.00	0		
*Actinomycetales*	Myc2	0.05	5	0.00	0		
*Actinomycetales*	Myc3	0.02	5	0.00	0		

**Fig 3:**
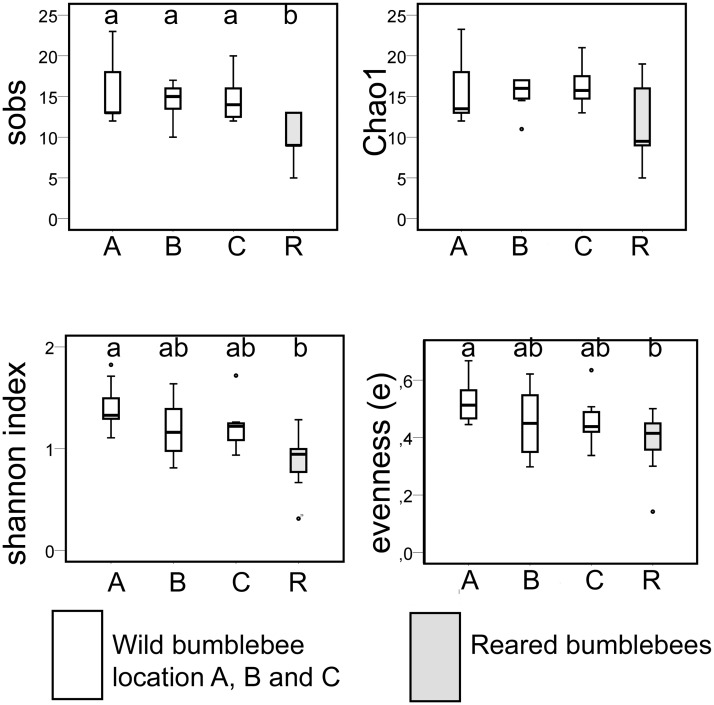
The observed richness (Sobs), Chao1 estimator, Shannon index and evenness (e) boxplots of wild specimens collected at location W1, W2 and W3 (in white), while indoor-reared bees are represented as R (in grey). Differences by pairwise post-hoc Kruskal-Wallis tests are indicated by a and b (P adjusted < 0.05). The boxplot shows the upper and lower quartiles and whiskers represent the range excluding outliers represented by **o**.

We calculated some basic parameters to describe the community richness (sobs = the observed richness and chao = Chao1 estimator), community diversity (the Shannon index), and community evenness (e) (Fig 3). The indoor-reared bumblebee specimens had lower parameters (sobs: Kruskal-Wallis χ2(3) = 16.5, *P =* 0.01; Chao1: χ2(3) = 7.3, *P =* 0.06; Shannon index: χ2(3) = 19.0, *P <* 0.001; and e: χ2(3) = 10.9, *P =* 0.013). The lower number of bacterial OTUs in each specimen does not result in a change in relative abundance of bacteria. Only for *Snodgrassella* an increase of its relative abundance was observed (see Fig 2).

The non-metric multidimensional scaling with similarity matrix overlay (Fig 1) showed that the microbiota of all indoor-reared bumblebees, except one outlier, have 50% similarity. As reported above the indoor-reared bumblebees indeed have a different bacterial composition which is confirmed by ANOSIM (R = 0.24, *P* = 0.002). The microbiota of indoor-reared bumblebees is mainly composed of the core-bacteria and *Bifidobacterium* (Bifido 3) (Fig 3). The outlier is characterized by the presence of a *Bacteroidetes* bacterium. Especially the *Enterobacteriaceae* have a different relative abundance between indoor-reared and wild bumblebees (Fig 2).

### Association between *Lactobacillaceae* and *Bifidobacteriaceae*

**Fig 4:**
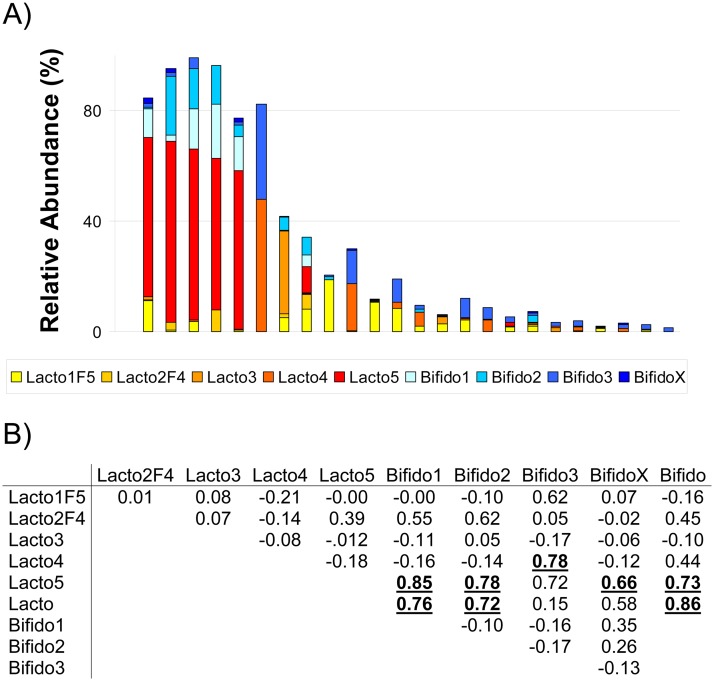
A) The normalized relative abundance (%) of the different *Lactobacillaceae* and *Bifidobacteriaceae* in wild *Bombus terrestris*. Specimens are ranked from high total *Lactobacillaceae* to low. B) Pairwise Pearson correlation coefficients, those in bold and underlined have *P* value below 0.00017.

An association study between the OTUs present in wild bumblebees revealed several associations between OTUs of the *Lactobacillaceae* and the *Bifidobacteriaceae*. These associations were not found in the intensively indoor-reared bumblebees. We used a strong Bonferroni correction resulting in a corrected Alpha of 0.00017 (Fig 4b). Mainly the presence of Lacto5 resulted in a higher relative abundance of different bifidobacteria as represented in Fig 4a.

## Discussion

### The new core bacteria

Deep sequencing of the microbiota of *B*. *terrestris*, one of the most common bumblebees in Europe, revealed several bacterial taxa known to be associated with corbiculate bees. *Snodgrassella*, *Gilliamella*, Lacto1-Firm5 and Lacto2-Firm4 have been described as core bacteria of *Apis* [34] and the former three are also quite prevalent in *B*. *terrestris* (see Table 3). The other OTUs, Bifido1, 2 and 3, Lacto3, 4 and 5, and *Bacteroidetes*, are known to be associated with honeybees and bumblebees but with a more erratic occurrence [15, 16]. Cariveau et al. [13] proposed a division in core and non-core microbiota, which can be useful to understand and describe the functionality of the microbiota. Core bacteria are repeatedly associated with individuals of a particular host species or cluster of closely related hosts. If we include deep sequencing data to further ameliorate this subdivision, then bifidobacteria can also be regarded as core bacteria in *B*. *terrestris*, with the Bifido3 OTU as the most prevalent one. Indeed bifidobacteria have a low relative abundance, but a high prevalence, with only 2 out of the 24 wild specimens having no OTU belonging to the *Bifidobacteriaceae* (data not shown). Our results confirm that *Lactobacillaceae* are core bacteria of *B*. *terrestris* (23 out of 24 specimens contain lactobacilli, data not shown), with Lacto1-Firm5 as the most prevalent OTU, while the other lactobacilli have a more sporadic occurrence (Table 3).

The OTUs Gamma-E1, Gamma-E2, Gamma-P, Firm-S, Firm-E, Burk1, Burk2 and *Actinomycetales* were confirmed to be present in our samples by OTU specific primers. Sanger sequence confirmation was performed because these bacterial sequences were not yet reported by non-deep sequencing studies. For the latter 5 of these 7 OTUs, the non-detection in previous studies can be explained by the deeper sequencing power of Illumina sequencing. Also for some of these OTUs similar sequences have been found in bumblebee specimens (see table 2). The Gamma-E1 and Gamma-E2 OTUs have a very high relative abundance, which likely should have been picked up during previous sequencing efforts (e.g. [15, 16, 35]). Bias in clone library construction and PCR amplification could explain why these sequences have remained undetected by non-deep-sequencing techniques. The detection of Gamma-E1 and Gamma-E2 is not a local phenomenon, as different *Enterobacteriales* were also present in the 454-sequencing data set of three North American bumblebee species[13]. We therefore argue to regard the yet to be specified genera or bacterial species within the family of *Enterobacteriaceae* as core gut bacteria. Indeed they can be the dominant OTU within the gut microbiota of wild *B*. *terrestris*, although remain undetected in the indoor-reared bumblebees. However their prevalence in bumblebees remains somewhat erratic. This suggests that the environment or other host genetic or physiological parameters could be more important for their presence. Cariveau et al. [13] reported a negative association of *Enterobacteriales* presence in the gut and *C*. *bombi* infection. Aside from this, *Enterobacteriales* have been reported to have a nitrogen fixation function in the fruit fly *Ceratitis capitata* [36], and they have been found in different beetles and their larvae where the importance of this bacterial family for concentrating nitrogen for the developing larvae has been debated [37–39]. For now their role is somewhat ambiguous, but a potential nutritional role should be investigated further.

### The indoor-reared bumblebee has a subset microbiota

To date *B*. *terrestris* are reared in a closed intensive breeding system and so commercially used for biological pollination [3]. Within such a system the ability for horizontal transmission of bacteria is impaired. During colony development nutrition is deposited inside the nest and foragers are unable to leave the nest. Bacterial transmission is only possible between nests in close proximity of each other. Horizontal transmission is still possible when the queens are released for their mating flight in order to ensure a new breeding stock. But the loss of contact with outside bees and flowers could induce a bottleneck in the microbiota of indoor-reared bumblebees. Indeed the microbiota of indoor-reared *B*. *terrestris* is a subset of its wild microbiota. There are two bacteria which we did not find in the wild bumblebees: Firm-B occurred in two bees, while Gamma-2 was found in one reared bee. Gamma-2 has also been described as a core-bacterium, mainly because of its presence in honeybees [34], but it is rather scarce in bumblebees, including *B*. *terrestris* [15, 40], and therefore we consider it as the non-core sister of *Gilliamella*. Firm-B can be considered as a non-core bacteria, previously identified with culture dependent techniques in reared blackened bumblebee larvae [41].

The NMDS plot (Fig 1) demonstrated that all intensively reared bumblebees had a similar microbiota, with only one specimen falling outside this group, a specimen having *Bacteroidetes* with a high relative abundance of 93%. All other specimens were dominated by *Snodgrassella* and *Gilliamella*. Also Lacto1-Firm5, Lacto2-Firm4 and Bifido3 were present in indoor-reared bumblebees (Table 3). It seems plausible that these bacteria have the potential of vertical transmission (be it with or without the means of contact of two generations within one colony); while for the others (mainly non-core bacteria) horizontal transmission routes from the environment might be more important. However this hypothesis remains to be tested.

The lack of absolute numbers of bacteria restrains us to make informed decisions on the actual bacterial abundance. It remains possible that wild samples harbor low bacterial titer of *Snodgrasella* and *Gilliamella* compared to reared bumblebees, and therefore more exotic bacteria could be detected in wild bumblebees. Therefore it would be interesting to check for correlations between the abundance of certain bacterial taxa and the absolute titer of the total gut microbiota.

The present data demonstrate that intensively indoor-reared bumblebees cannot be regarded as harboring a wild microbiota, as they have a lower bacterial diversity (Fig 3) and a higher relative abundance of *Snodgrassella* (Fig 2). Indoor-reared bumblebees are however useful as a simplified model for the microbiota of wild bumblebees which allows to study the interaction of *Snodgrassella*, *Gilliamella*, *Lactobacillus* and *Bifidobacterium*, in a setting with minimal biological variation. This is ideal as a first step of hypothesis testing. The use of indoor-reared bumblebees makes the study of bacterial dynamics and interactions in relation with age, nest development, or caste more feasible.

### What about bacterial spillover?

Aside from the fact that indoor-reared bumblebees harbor a core set of bacteria known to be host-associated, our results also showed they lack bacteria not known to be associated with bumblebees. Therefore when indoor-reared bumblebees are placed outside for biological pollination purposes, they will not directly spread non-host associated bacteria and thus will not act as a driver of dysbiosis in wild bumblebees. This mechanism of spillover has been described for parasites of managed bees. Indeed domesticated honeybees [42] or reared bumblebees [43] can spread parasites and thereby negatively influence the already endangered status of many wild pollinators [44].

Although we do not see a dysbiosis in indoor-reared bumblebees, it remains to be investigated if the microbiota changes when the bees are placed outside for their pollination purpose and if the microbiota is suited to prevent viral or parasite infection, like reported for wild bumblebee microbiota[13, 15].

### Gut colonization of *Bifidobacteria*

Another striking observation was the positive association between *Lactobacillus* and *Bifidobacteria*. This general association in our data exists because of a specific association of Lacto5 with Bifido1, Bifido2 and BifidoX, and of Lacto4 with Bifido3. The association of these specific OTUs is not a consequence of them being present at only one location, as Lacto5 is retrieved from bumblebee samples in all locations. A possible explanation for the associations is that certain lactobacilli are needed to create a suitable environment promoting the growth of bifidobacteria. Studies on human gut colonization dynamics revealed that lactobacilli, among others, are initial colonizers. They are facultative anaerobes and thus reduce the oxygen levels enabling the growth of anaerobic bifidobacteria [45]. This common mechanism of oxygen deprivation can however be performed by a vast majority of the bacteria present in the bee gut. Therefore the specific correlation does not need to be a strict reliance on each other, it could be that the combined drop of certain gut core bacteria allowed for a better relative detection of low abundant bacteria. The observed correlation is indeed between two non-core lactobacilli and the low abundant bifidobacteria.

## Supporting Information

S1 Dataset List of all OTUs before applying the criterion of 0.5% prevalence of an OTU in at least one specimen. (XLS)

S2 Dataset List of OTUs and their representative sequence. (XLS)

S1 Fig The residual versus fits plot and the mean-variance plot of the multivariate bacterial relative abundance data. (TIF)

S2 Fig A) The number of sequences needed per specimen: the rarefaction curve shows the mean numbers of OTUs per location or breeding facility in function of the reads per specimen (sample). B) The number of specimens needed per location: the rarefaction curve shows the numbers of OTUs per location or breeding facility in function of the numbers of specimens analyzed.
(TIF)

S1 Table Wald-score after the anova.manyglm function within the mvabund package in R of the normalized abundance of all OTUs comparing bumblebees from three wild location and indoor-reared specimens. (DOC)
