# Supplementary figures and images for: 16S rRNA Amplicon Sequencing Demonstrates that Indoor-Reared Bumblebees (Bombus terrestris) Harbor a Core Subset of Bacteria Normally Associated with the Wild Host
Source: PLoS One. 2015 Apr 29;10(4):e0125152. doi: 10.1371/journal.pone.0125152 (PMC4414509; doi:10.1371/journal.pone.0125152)

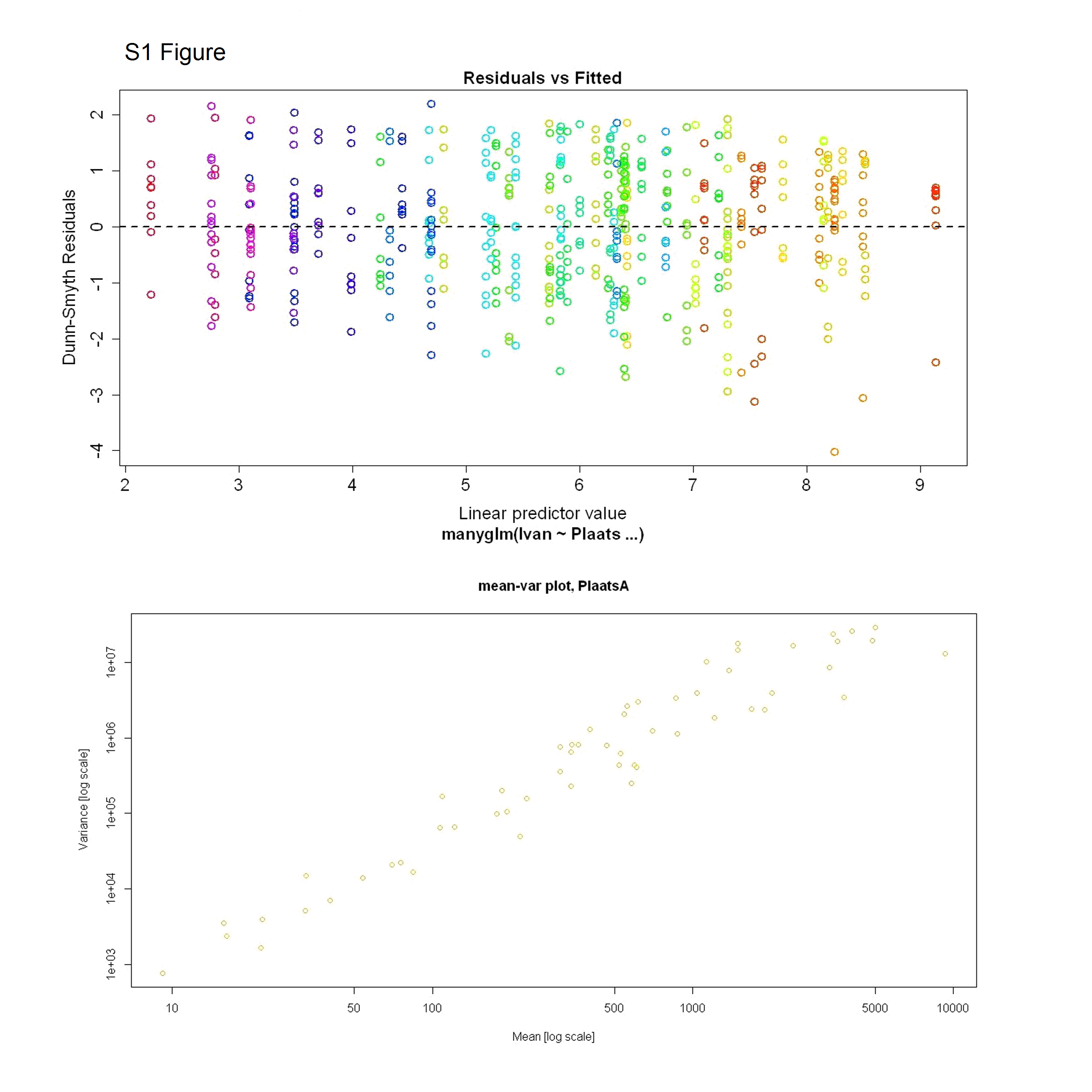

Supplement: S1 Fig — (TIF) [file pone.0125152.s003.tif]

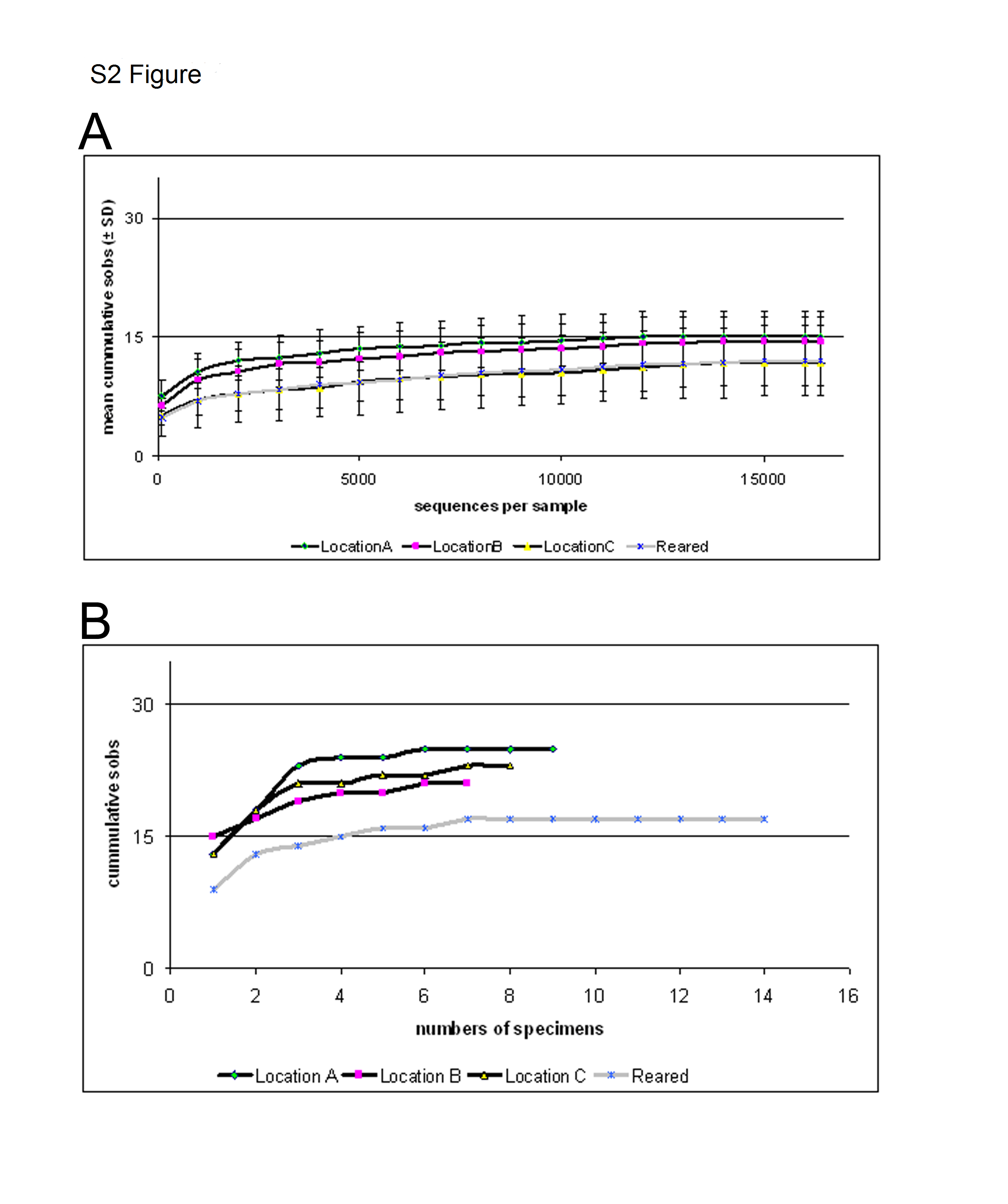

Supplement: S2 Fig — A) The number of sequences needed per specimen: the rarefaction curve shows the mean numbers of OTUs per location or breeding facility in function of the reads per specimen (sample). B) The number of specimens needed per location: the rarefaction curve shows the numbers of OTUs per location or breeding facility in function of the numbers of specimens analyzed. (TIF) [file pone.0125152.s004.tif]
