# Supplementary material for: 16S rRNA Amplicon Sequencing Demonstrates that Indoor-Reared Bumblebees (Bombus terrestris) Harbor a Core Subset of Bacteria Normally Associated with the Wild Host
Source: PLoS One. 2015 Apr 29;10(4):e0125152. doi: 10.1371/journal.pone.0125152 (PMC4414509; doi:10.1371/journal.pone.0125152)
Supplement: S1 Table — (DOC) [file pone.0125152.s005.doc]

S1 Table: Wald-score after the anova.manyglm function within the mvabund package in R of the normalized abundance of all OTUs comparing bumblebees from three wild location and indoor-reared specimens.

| **Multivariate test** |  |  |
| --- | --- | --- |
| Df. | Dev. | P-value |
| 34 | 173.6 | **0.001** |
| **Univariate test** |  |  |
|  | Wald-score | P-value |
| Snodgrasella | 9.975 | 0.135 |
| Gilliamella | 2.959 | 0.938 |
| Lacto1-Firm5 | 0.722 | 0.938 |
| Lacto2-Firm4 | 6.791 | 0.344 |
| Bacteroidetes | 0.827 | 0.938 |
| Bifido3 | 2.643 | 0.938 |
| BifidoX | 3.127 | 0.938 |
| **Gamma-E1** | **24.262** | **0.002** |
| **Gamma-E2** | **19.314** | **0.005** |
| Lacto5 | 8.146 | 0.233 |
| **Lacto4** | **14.456** | **0.027** |
| **Bifido2** | **12.652** | **0.041** |
| Bifido1 | 6.621 | 0.344 |
| Firm-S | 7.654 | 0.246 |
| Firm-E | 9.788 | 0.138 |
| Lacto3 | 7.739 | 0.241 |
| Myc 1 | 6.378 | 0.346 |
| Myc 2 | 6.372 | 0.346 |
| Gamma-P | 6.382 | 0.344 |
| Burk1 | 3.287 | 0.938 |
| Burk2 | 3.287 | 0.938 |
| Myc 3 | 3.026 | 0.938 |
| Alpha1 | 3.025 | 0.938 |
| Gamma-2 | 2.073 | 0.938 |
| Firm-B | 2.075 | 0.938 |
